# A rare late progression form of Sly syndrome mucopolysaccharidosis

**DOI:** 10.1002/jmd2.12039

**Published:** 2019-07-29

**Authors:** Nathalie Guffon, Roseline Froissart, Alain Fouilhoux

**Affiliations:** ^1^ Centre de Référence des Maladies Héréditaires du Métabolisme, Hôpital Femme Mère Enfant Hospices Civils de Lyon Bron France

**Keywords:** case report, mucopolysaccharidosis, Sly syndrome

## Abstract

Mucopolysaccharidoses VII, or Sly syndrome, is linked to mutations in the beta‐glucuronidase encoding gene. Sly syndrome is a rare condition and presentation is highly variable, ranging from a prenatal form with severe, lethal fetal hydrops to more benign adolescent or adult forms with simple thoracic kyphosis. Molecular diagnosis of this adult male patient identified two missense mutations in the *GUSB* gene that led to a deficiency in beta‐glucuronidase catalytic activity and the resulting accumulation of chondroitin sulfate glycosaminoglycans. During childhood, bilateral inguinal hernia was repaired at 1 year of age and gait abnormalities were noted, leading to a bilateral femoral varization osteotomy due to a bilateral coxa valga with hip subluxation at the age of 7.5. The patient suffered regular upper respiratory infections and required numerous orthopedic surgeries. Despite learning difficulties with visual and hearing deficits, the patient worked full‐time and undertook regular leisure activities. At 33 years of age, the patient's health deteriorated; a hip replacement and glaucoma leading to reductions in his visual field limited his capacity to travel independently. The patient was hospitalized at 51. Although he remained self‐sufficient for taking meals, he needed help with many daily activities. Following a period marked by major asthenia with a general loss of autonomy, the patient died at 52 years of age. With the advent of new enzyme replacement therapies, this medical history of this rare untreated attenuated patient may provide benchmarks to judge the efficacy of treatment in future patients.

## 1 INTRODUCTION

Mucopolysaccharidoses (MPSs) are lysosomal storage disorders caused by the deficiency of an enzyme responsible for the degradation of mucopolysaccharides or glycosaminoglycans (GAGs). Distinct forms of MPS are each associated with a particular enzyme deficiency, seven have been described to date. With the exception of X‐linked MPS‐II, MPSs are autosomal recessive.

Sly syndrome, or MPS‐VII, is specifically linked to a mutation in the beta‐glucuronidase (*GUSB*) gene located on the long arm of Chromosome 7 (7q21‐q22), more than 45 types of mutations have been identified.^1^ *GUSB* is involved in the degradation of several GAGs that are constituents of connective tissue, including dermatan sulfate (DS), heparan sulfate (HS), and chondroitin sulfate (CS).^2^ Its deficiency causes their accumulation in the lysosomes of various tissues and organs, including the central nervous system, resulting in multisystem damage.

Sly syndrome is rare, with an estimated frequency of 1:300,000 to 1:2,000,000.^3^ The phenotype is variable, ranging from a prenatal form with severe lethal fetal hydrops to benign adolescent or adult forms with simple thoracic kyphosis. Severity can be attributed to genotype and residual enzymatic catalytic activity. However, the extremely variable presentation of this disease, which can progressively affect many organ systems, renders early diagnosis difficult on clinical grounds alone in moderate cases. Mucopolysacchariduria, that is, increased levels of urinary GAG (CS alone or DS, HS, and CS combined) guide the diagnosis; however, in moderate clinical forms, especially in adults, the urinary GAG levels may be normal or only slightly elevated. Granulocyte studies may reveal coarse metachromatic inclusions in these patients.

Diagnoses are confirmed by the demonstration of a beta‐glucuronidase deficiency in leukocytes or fibroblasts, but prognosis remains unclear. Enzymatic activity levels do not enable an accurate determination of disease severity. Molecular genetic tests for mutations in the *GUSB* gene are also available to confirm the diagnosis and may sometimes provide more insight into prognosis. Prenatal diagnosis is possible by amniocentesis or chorionic villus sampling to measure beta‐glucuronidase activity or via molecular genetic testing for mutations in the *GUSB* gene.

This rare case of MPS‐VII has atypical features that highlight its distinctive manifestation of the attenuated, late progression form of Sly Syndrome, and its evolution over a lifetime.

## 2 CASE PRESENTATION

The MPS‐VII diagnosis of this male patient was made at the age of 22.5 years, based on (a) the presence of a high level of urinary GAG (64.4 mg/g creatinine) with the exclusive detection of CS, (b) a deficiency in beta‐glucuronidase activity in leukocytes (3.9% of normal controls), and (c) the identification of two missense mutations in the *GUSB* gene, p.R382H (c.1145 G > A) and p.Y508C (c.1523 A > G), as previously reported.^4^

No familial antecedent was reported among the four brothers and healthy, nonconsanguineous parents. Born at term after a normal pregnancy, with a normal neonatal exam and a high weight (4 kg), surgery for bilateral inguinal hernia was required at 1 year of age. He also presented recurrent upper respiratory infections with a speech delay (first words at 18 months). First noted at 6 years of age, gait abnormalities led to a hospitalization for neurologic evaluation, radiological exams, and orthopedic advice. At the age of 7.5, he received a bilateral femoral varization osteotomy due to bilateral coxa valga with hip subluxation. The diagnosis was that of spondyloepiphyseal dysplasia associated with growth retardation and moderate dysmorphia. A subsequent growth retardation led to the diagnosis of dwarfism at 10, with a final height of 135 cm at 16 years old.

Schooling was problematic due to learning difficulties and attention disorders. However, from 14 to 19 years old, the patient was cared for in a medical‐educational institute and then worked in a Center for Help by Work. From the age of 33, the patient was limited to part‐time work due to his deteriorating state of health, but did maintain weekly leisure activities.

His initial clinical follow‐up was essentially orthopedic with a childhood marked by numerous surgeries. At the age of 10, he suffered a recurrence of left hip subluxation and complete dislocation of the right hip that required right‐sided Colonna's intervention, followed a year later by left‐sided osteoplasty. He then presented an evolutive and aggravated genu valgum, treated at 14 years by an internal epiphysiodesis of the inferior conjugation cartilage of the two femurs. However, the persistence of the asymmetric genu valgum, predominant on the left, required the use of orthopedic shoes with stakes and two canes, or the use of a wheelchair over longer distances. At age 15, worsening of dysmorphia, thoracolumbar kyphosis, muscle deficit, sharp osteotendinous reflexes, limitation of shoulder elevation, and very lax knees were noted. Since 18 years of age, a worsening of the genu valgum, especially on the left side, was noticed and led to a high tibial corrective osteotomy at 22 years.

With regard to otorhinolaryngology, three adenoidectomy were performed, the last at the age of 14, along with tonsillectomy to prevent recurrent ear infections. The mean mixed hearing loss was diagnosed at age 13 with the loss of 40 dB on the left and 50 dB on the right. As a result, he had a delayed language development and used lip reading.

Ophthalmologically, small corneal opacities were evident at 14 years of age, visual acuity at 15 years was 5/10 in the right eye and 9/10 in the left eye, then 7/10 right and 7/10 left at 18 years.

At 22, it was his rehabilitation center's doctor who tested for MPS. The hip bone progressively deteriorated (no extension, 80° flexion on the right and 70° on the left, 40° abduction on the right and 50° on the left, adduction 20°, loss of internal rotation, and external rotation 30°) with significant pain in the right hip, and shortening of 3 cm of the left lower limb. Total hip replacement was performed at 29, under slightly difficult conditions (thick tissues and articular capsule). Intraoperatively, total necrosis of the femoral head was observed along with a very small diameter of the femoral neck. The diameter was ~18 mm, which caused difficulty in placing even a small‐size prosthesis. The acetabulum had a very good appearance, and allowed the seating of the cup. Subsequently, the patient presented with an aggravation of his leg length inequality, with the right leg being 5 cm longer. For this, he received orthopedic shoes with an initial left‐side compensation of 3 cm and then 4 cm.

At the age of 33, a sudden deterioration was reported with pain during walking and support. X‐rays showed complete loosening of both the stem and the cup. A change of the right prosthesis was performed in the same year. This procedure was very complicated because of the particular morphology of the femur and pelvis and poor bone quality. In the end, it was possible to introduce an unsealed total right hip prosthesis and an acetabular screwed ring. Intraoperative, it was found that the acetabulum was completely loosened, which resulted in a significant loss of bone mass in the acetabular fossa and especially the acetabular lip, which required an allograft. At the time, the left hip was still dislocated but painless and maintained good mobility. Following this prosthetic change, the patient had been able to walk with two elbow crutches.

Concerning the interoperative follow‐up, retinitis pigmentosa with bone spicule intraretinal pigment formations were detected by fundoscopy at the age of 36. At the age of 40, the patient was diagnosed with glaucoma, which was treated with eye drops. In addition, he presented with a restriction of his visual field, difficulties of adaptation to shadows, and difficulties in particular to discern uneven surfaces such as on sidewalks. Corneal opacities worsened, especially after the age of 40 and bilateral cataracts were noted at the age of 43.

At 50, the patient was hospitalized for polymicrobial sepsis (*Streptococcus constellatus*, *Bacteroides fragilis*, then pyocyanin) whose point of entry was an abscess complicating a diverticular sigmoiditis. The patient had no history of digestive disorder. The evolution was favorable under antibiotic therapy (clamoxyl and gentamicin, then piperacillin, ciflox, and flagyl). However, the infection was complicated by portal thrombosis leading to treatment with previscan. On this occasion, transthoracic echocardiography to assess the presence of infectious endocarditis revealed aortic insufficiency. A subsequent echocardiography, performed a few months later when the patient was in a better condition, confirmed Grade I‐II aortic insufficiency, a tight supravalvular aortic stenosis (mean left ventricle [LV]‐aortic gradient 47 mm Hg; max 73 mm Hg; functional aortic surface area 0.5 cm^2^), left ventricular hypertrophy without intraventricular gradient, and minimal mitral insufficiency in an unexpanded left atrium. The LV function was retained (ejection fraction 64%), the LV was not dilated. The electrocardiogram performed at the same time was normal. The patient was therefore treated with betablocker. In addition, the patient began a treatment for pseudo‐asthma (an inhaler of corticoids and bronchodilator).

**Figure 1:**
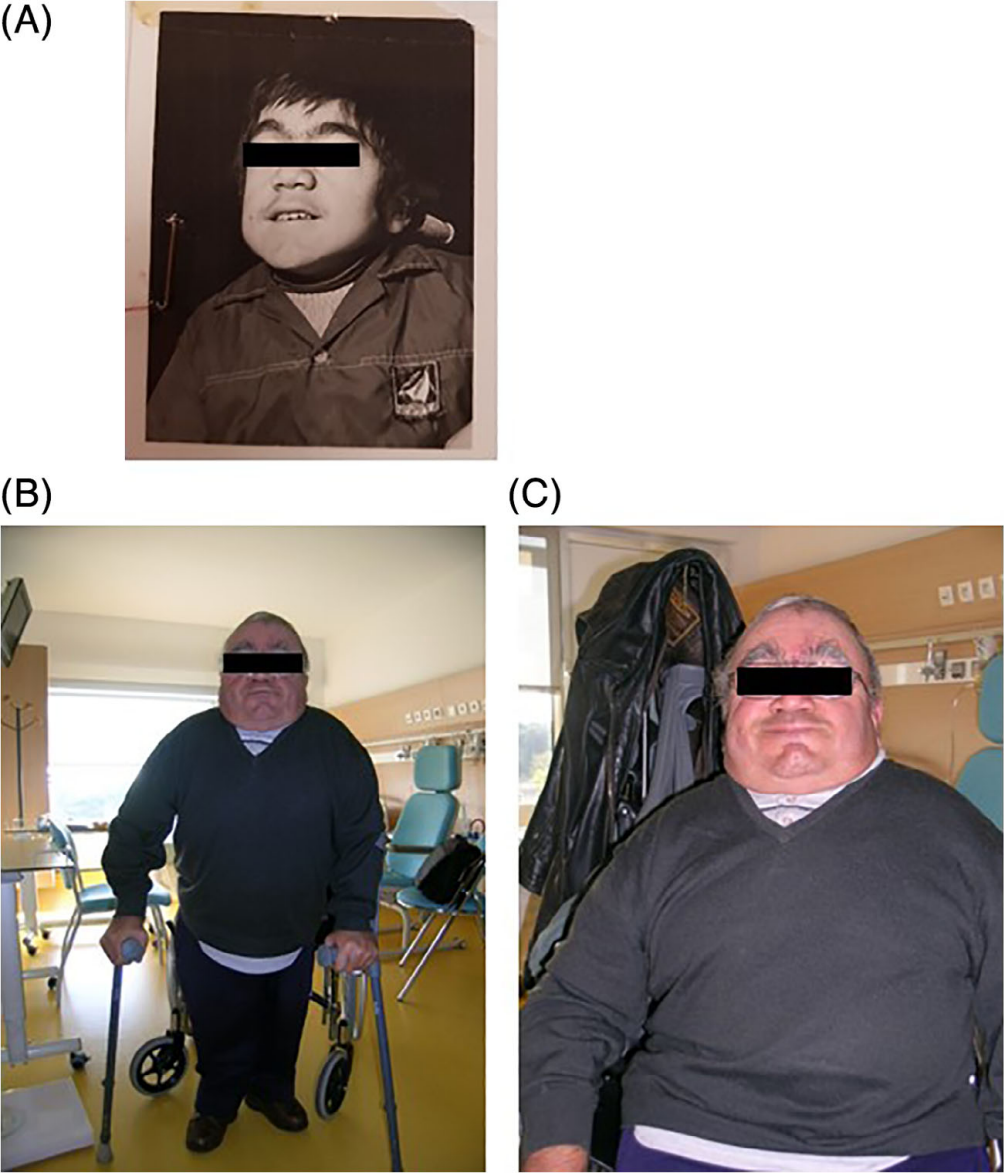
Photographs of the patient at 14 years (a) and 51 years of age (b and c)

The patient accepted hospitalization for a complete evaluation when he was 51 years old, in light of his worsening clinical status. He weighed 62 kg with a height of 130.5 cm (see photographs of the patient in Figure 1). He was receiving weekly physiotherapy and the intervention of a nurse's aide twice a week. Although self‐sufficient for taking meals, he needed help with daily activities (ie, putting on shoes and socks, combing hair, washing, and pouring a glass of water). Walking was difficult with two crutches, requiring the help of a third person. Climbing stairs was very difficult and he was unable to get on and off a bus alone. He could not bend over or pick up an object that had dropped to the ground, and standing up from a chair or the toilet was also difficult. The patient stated that in daily life the joint injuries in his shoulders, hips, knees, and ankles were a source of embarrassment. He had no complaints of pain, and slept 12 hours per night. There was night snoring without sleep apnea, as reported by the family. Incidents of somnolence sometimes occurred before meals. There was no presentation of headache. His treatment regimen at that time included a betablocker, anti‐vitamin K, and a corticosteroid and bronchodilator inhaler. The detailed clinical assessment and management of the patient in our unit are summarized in Table 1.

**Table 1:** Description of the complete medical condition during his follow‐up at our service at the age of 51

Exam	Results
Physical examination	Pulmonary auscultation is normal. The patient is unable to perform a pulmonary function test. Cardiac auscultation regains a systolic murmur. The abdomen is flexible. There is no organomegaly. There is no hernia. Osteotendinous reflexes are weak in the lower limbs, normal in the upper limbs. There is no epileptoid trepidation. There is no Babinski sign. There is thoracolumbar kyphosis. The movements of the elevation of the shoulders are limited. The hands are in claw. The joints of the elbows and wrists are rather supple. The joints of the lower limbs are quite flexible. There is an asymmetric genu valgum on the right side
Biology report	Beta‐d‐glucuronidase activity: In leukocytes: at 6 μkat/kg (3.9% of normal control) In fibroblast: 1.1 μkat/kg (1.7% of normal control) In serum: not detectable Normal net filtration pressure Normal ionogram Normal liver assessment Normal thyroid balance Urinary excretion of glycosaminoglycans at 27.7 mg/g creatinine with significantly high excretion of CS and traces of HS and DS *GUSB* gene: heterozygous composite for mutations p.R382H (c.1145 G>A) and p.Y508C (c.1523A>G)
Abdominal scan	No perisigmoid abscess but persistence of an important infiltration of the perisigmoid fat and multiple diverticula and supracentimetric ganglia in contact with the large vessels. Portal thrombosis with presence of a cavernoma. The thrombosis extends to the splenic vein and mesenteric vein. No hepatosplenomegaly. Homogeneous liver. Kidneys of normal size, well differentiated, undiluted. Large cyst (11.6 cm) on the left kidney containing fine, hyperdense septa. No intraperitoneal effusion. Aplasia of the left femoral head
Orthopedic radiological assessment	Total hip prosthesis right, no wear of the acetabulum. On the other hand, kyphosis centered on T12 by necrosis and collapse, thoracic lordosis above. Genu valgum asymmetrical on the right side. *Management*: no correction of thoracolumbar hyperkyphosis, given his state of health; maintain both elbow crutches and continue physiotherapy twice a week
Cardiological assessment	Clinically: systolic murmur 2.5/6 Normal echocardiogram Echography: mitro‐aortic dystrophy with severe aortic stenosis and aortic regurgitation 1.5/4 and mitral regurgitation 1/4. No mitral stenosis. Symmetric left ventricular hypertrophy (IVS 13 mm, PP 13 mm) with normal left ventricular function (EF = 67%). Theoretical indication of aortic valve replacement, but this remains unfeasible due to the comorbidities *Management*: Prophylaxis of infectious endocarditis and continuation of vitamin K antagonist were indicated
ENT and audiophonology	Significant hearing impairment The auditory assessment indicates a bilateral hearing loss, but more so on the right side with a discrete transmissivity factor, which is entirely a matter of fitting of the hearing aid *Support*: Implementation of auditory equipment was very well accepted with a permanent port and a clear improvement of its intelligibility and understanding
Ophthalmology	Visual acuity on the right of 1.5/10 Parinaud 8 and on the left of 2.5/10 Parinaud 8 with correction Ophthalmologic complications with diffuse bilateral corneal opacities, glaucoma, and retinopathy pigmentosa *Management*: Intraocular tension normally maintained through hypotonic eye drops

Abbreviations: CS, chondroitin sulfate; DS, dermatan sulfate; HS, heparan sulfate.

At the age of 52, a worsening of the general condition and in particular respiratory function was reported with a major asthenia, gradually followed by a loss of autonomy, a loss of landmarks in time and space along with a haggard look. There were reports of uncontrolled movements of the legs and arms, especially in the morning, without loss of consciousness. The patient died 1 month later.

## 3 DISCUSSION

Born in 1958 prior to the initial characterization of the disorder by Sly et al.,^5^ this patient with an atypical clinical course did not benefit from molecular diagnosis until 22 years of age.

While attenuated forms of MPS‐VII have been reported, in most cases patients present with hydrops fetalis at birth and fail to survive beyond the first months of life.^6^ Sufficient residual enzyme activity, as little as 1.4% that of normal, may be sufficient to reduce disease severity.^7^ Although no residual activity was found upon testing this patient's serum, the beta‐glucuronidase activity detected in leukocytes and fibroblasts may explain the late progression of the disease in this case.

Presentation of MPS‐VII in those patients that survive past infancy remains variable,^7^, ^8^ with severity being linked to the specific mutation harbored by each patient.^6^ Genetic testing of this patient identified two substitution/missense mutations. Expression studies of the mutant enzymes were performed and led to a marked reduction of beta‐glucuronidase activity (2.3% and 19% of wild type for mutations p.R382H [c.1145 G>A] and p.Y508C [c.1523 A<G], respectively).^9^ These mutations affect amino acids that are highly conserved between species and that have both been predicted to be damaging by in silico studies using three bioinformatics tools (SIFT, Mutation Taster, and PolyPhen).

Survival data for this very rare disease are scarce, a recent cross‐sectional analysis from 2017 presented clinical data from only 53 MPS‐VII patients; the median estimated age of survival for postnatally diagnosed MPS‐VII patients was 30 years.^7^ At 52 years old, the patient presented here largely surpassed this estimated life expectancy, as well as that of reported individual cases such as the patient described in Storch et al.^10^ that had survived to age 37. The patient did not have a history of hydrops fetalis, the most frequent single distinguishing feature of MPS‐VII, but his medical history began with an operation to correct bilateral inguinal hernia at 1 year of age, as well as other typical clinical signs of more attenuated forms of MPS‐VII including short stature, skeletal dysplasia, hearing loss, cognitive impairment, and cardiac involvement.^7^, ^8^

In five cases of bone marrow transplantation treatments reported in MPS‐VII, their success was limited.^7^, ^8^ A novel enzyme replacement therapy has recently been described that may provide another curative treatment option for future patients.^11^ In this case, we have reported a patient whose symptomatic treatment history and special education opportunities enabled him to maintain employment in a Work Assistance Establishment into his 30s, as well as weekly leisure activities; part‐time employment until the age of 52. Should enzyme replacement therapy improve MPS‐VII life expectancies, this exceptional medical history offers insights to guide future treatments to improve the quality of life of these rare patients.

**Abbreviations**
CS: chondroitin sulfate
DS: dermatan sulfate
GAG: Glycosaminoglycan
HS: heparan sulfate
LV: left ventricle
MPS: mucopolysaccharidosis

## AUTHOR CONTRIBUTIONS

N.G., R.F., and A.F. participated in the assembly of this patient's medical history and its presentation herein.

## CONFLICT OF INTEREST

The authors declare no potential conflict of interest.

## ETHICS APPROVAL

This retrospective study presents the medical history of one patient. All procedures followed were in accordance to the ethical standards described in the Helsinki Declaration of 1975, and revised in 2000. As per local French legislation, no additional ethics committee approval was required for this report. This article does not include any studies with animal subjects performed by any of the authors.

## INFORMED CONSENT

The patient presented here has deceased, without leaving any specific interdiction of sharing his medical records. His family have been informed and provided their consent for the publication of this case report, in line with current French legislation.
